# Exploring Social Media Recruitment Strategies and Preliminary Acceptability of an mHealth Tool for Teens with Eating Disorders

**DOI:** 10.3390/ijerph18157979

**Published:** 2021-07-28

**Authors:** Erin Kasson, Melissa M. Vázquez, Christine Doroshenko, Ellen E. Fitzsimmons-Craft, Denise E. Wilfley, C. Barr Taylor, Patricia A. Cavazos-Rehg

**Affiliations:** 1 Department of Psychiatry, Washington University School of Medicine, St. Louis, MO 63110, USA; erinmkasson@wustl.edu (E.K.); melissavazquez@wustl.edu (M.M.V.); christine.xu@wustl.edu (C.D.); fitzsimmonse@wustl.edu (E.E.F.-C.); 2 Department of Psychology, Washington University in St. Louis, St. Louis, MO 63110, USA; wilfleyd@wustl.edu; 3 Department of Psychiatry and Behavioral Sciences, Stanford University School of Medicine, Stanford, CA 94305, USA; btaylor@stanford.edu; 4 Center for m2Health, Palo Alto University, Palo Alto, CA 94304, USA

**Keywords:** eating disorders, teens, adolescents, social media, mHealth

## Abstract

(1) Background: The current study leveraged social media to connect with teens with EDs to identify population specific characteristics and to gather feedback on an mHealth intervention. (2) Methods: We recruited teens with EDs from social media in two phases: (1) Discovery Group, (2) Testing Group. The Discovery Group (*n* = 14) participants were recruited from Facebook/Instagram and were asked to review the app for up to one week and provide qualitative feedback. After incorporating feedback from the Discovery Group, we refined our social media outreach methods to connect with 30 teens with EDs to pilot this mobile app. Recruitment from a variety of platforms on social media was successful, with the majority of enrolled participants in the Testing Group coming from Snapchat (60%) and a large percentage of participants belonging to gender and sexual minority groups (63%). (3) Results: Participants from both groups experienced extremely high rates of depression (100% Discovery, 90% Testing) and/or anxiety symptoms (100% Discovery, 93% Testing) in addition to ED symptoms, and noted this as a possible barrier to app engagement. (4) Conclusion: Use of social media for recruitment of teens with EDs is feasible and may connect with groups who may be more difficult to reach using traditional recruitment methods. Among the Discovery Group there was high acceptability of and interest in an app to support ED recovery, and characteristics of both groups demonstrated need for support in other mental health domains. Future studies should evaluate the preliminary efficacy of such tools among teens to determine the effects of such interventions on ED symptoms and other mental health outcomes.

## 1. Introduction

Eating disorders (EDs) are serious mental illnesses that cause impairment in a number of different domains of physical and psychological health and quality of life [1]. EDs are associated with high mortality [2] and are often comorbid with a number of other psychiatric conditions, such as mood and anxiety disorders [3]. EDs also have one of the highest rates of medical complications among psychiatric disorders and put adolescents at increased risk for a number of physical problems [3,4]. EDs often begin during adolescence, and early intervention is crucial to improving outcomes, especially in developing youth. By age 6, girls especially are already expressing concerns about their shape and weight [5]. However, many teens are unlikely to seek treatment for their EDs for numerous reasons, including being fearful of disclosing ED symptoms to parents [6,7], not feeling as though the severity of their symptoms warrant treatment [6], or not being ready for in-person or professional treatment [8]. Only about 20% of teens seek professional treatment [9], placing those who do not receive intervention at risk for further progression of illness and poorer outcomes [10,11,12].

Research has shown that teens engaging with body image content on social media (SM) that idealizes thinness may be more likely to suffer from eating or body image concerns, and some studies have linked increased SM use with ED risk [13], pointing to the crucial role of this media in the formation of these concerns [14,15,16]. This is concerning, as 97% of teens report use of at least one SM platform, including YouTube, Instagram, Snapchat, Facebook, Twitter, Tumblr, or Reddit [17]. Further, 95% of teens report owning or having access to a smart phone daily [17]. As such, this interest in and ubiquitous use of social media and technology among teens could be leveraged to improve health outcomes, including mental health and ED symptoms.

There is support for the use of digital tools for individuals struggling with EDs [18,19,20]; however, fewer investigations have examined the use of such technologies to specifically support teens with EDs. Emerging literature suggests preliminary acceptability and efficacy of guided computer- and internet-based interventions among teens with bulimia nervosa (BN), yet results require confirmation in randomized controlled trials and further replication among teens with BN and other EDs [21,22,23]. Other work has shown that such digital health interventions aimed to reduce depression and anxiety show clinical benefit among teens, particularly those based on cognitive-behavioral therapy (CBT) principles [24,25], demonstrating cost-effectiveness in comparison to in-person treatment [26,27], moderate to high youth engagement [28,29], and a greater sense of control for some youth in managing difficult topics through technology than in person [30,31]. Digital intervention and outreach methods also enable researchers to reach a more demographically inclusive sample, including groups that may struggle with unique barriers to accessing treatment (i.e., males, gender and sexual minorities, and racial/ethnic minorities) [32,33,34]. In addition to attenuating structural barriers such as financial limitations, availability of providers, and transportation to care, digital interventions have the potential to overcome some attitudinal barriers toward ED treatment implicated in contributing to mental health disparities, like lack of perceived need, stigma/shame, distrust, and lack of cultural competency of providers [35,36,37,38,39].

Further research is needed on specific outreach methods using social media for teens at-risk for and experiencing ED symptoms and to determine ways in which to connect them to a tailored digital intervention meeting their unique needs [24,40,41]. Input from these target users is essential to create and adapt such mHealth interventions to be user-centered and applicable to the preferences of adolescents in need of ED recovery support to increase impact on outcomes and likelihood of sustained use [41]. As such, the current study recruited groups of teens in two phases to both (1) test innovative outreach methods on social media to connect with teens with EDs and to identify unique characteristics and risks within this target population to tailor outreach and intervention, and (2) gather preliminary feedback on an mHealth intervention designed to address body image and eating concerns among teens.

## 2. Materials and Methods

Teens were recruited in two phases during this study including the Discovery Group and Testing Group, as in line with similar frameworks to guide refinement of digital therapeutics [42].

### 2.1. Discovery Group Methods

For the initial Discovery Group, teens aged 14–17 years old who identified as female and who screened positive for ED symptoms or at high risk for an ED based on the Stanford-Washington University Eating Disorder Screen (SWED) were recruited from social media [43]. Advertisements on Instagram and Facebook and specifying keywords related to body image and eating concerns (e.g., weight, shape, thin, waist) informed by previous research were used to target outreach to this demographic group [44]. These two platforms were chosen for initial recruitment testing as Instagram is one of the most popular platforms among teens [17] and image-based social networking through such platforms has been found to be particularly related to body image and eating concerns [45,46,47,48]. Additionally, because Instagram is owned by Facebook, they share a common advertisement manager such that we could share the Instagram advertisements on Facebook simultaneously for the same price as Instagram alone. Once teens clicked on a targeted ad, they reviewed an online assent document, and after providing assent were then routed to a screening survey assessing age (by providing birth year), current ED treatment (eligible only if not in treatment), and ED symptoms using the SWED. If eligible, teens were asked to provide contact information and continued to the baseline survey. After completing the baseline survey, teens were sent an email invite to access the standard app (with content for body image and EDs previously developed and tested with adults) [19,49] and PDF copies of app content tailored for teens. Teens were asked to review app features and content in the standard app and to compare these with the proposed content changes for teens in the PDFs for up to one week. They were then provided a unique link to complete a survey including quantitative and qualitative questions to garner feedback on the program.

### 2.2. Testing Group Methods

Informed by number of teens reached and sample characteristics gathered from the Discovery Group, refinements were made in recruitment strategies for the Testing Group. Given that the Discovery phase demonstrated we were able to reach a high percentage of sexual minority teens through SM recruitment, recruitment was expanded in the Testing phase to see if this approach would also be able to reach other groups not traditionally included in ED treatment or who also experience unique barriers to traditional care (i.e., males and gender minorities). As such, inclusion criteria remained the same except for gender identity, which was removed from criteria for the Testing Group. Recruitment strategies were also expanded to include Reddit and platforms including Snapchat, TikTok, and YouTube in addition to Instagram and Facebook, in order to reach an even broader sample. The workflow from targeted ads, online assent, screener, and baseline survey were identical to the Discovery Group. Advertisements for the Discovery Group included static images, while advertisements for the Testing Group including static images, images with animation, and video advertisements. All three types of advertisements were used across all platforms for Testing Group recruitment, except for YouTube and TikTok which used only images with animation or video advertisements given the structure of content on these platforms. The screener was altered in the testing phase to move age exclusion to the end of the screen in order to gather additional information on different age groups reached by this social media recruitment approach (e.g., those 18–19 years old). After completing the baseline survey, Testing Group teens were sent an email invite to access the app with teen related adaptations informed by Discovery Group feedback previously garnered. Teens in the Testing Group were asked to engage with the app for 2 months. Email survey reminders for those who did not fully complete the baseline survey and email links to follow up surveys were sent to participants in both the Discovery Group and Testing Group to encourage study activity completion and retention.

Evaluations of pilot efficacy and usability of the refined mHealth tool are currently underway, and the current manuscript focuses on preliminary feedback on the initial tool and adaptations made for adolescents as well as refinements of social media outreach methods. All surveys from both groups were completed in Qualtrics. The Washington University Institutional Review Board granted a waiver of parental consent for this study due to the minimal risk involved (IRB # 201911015).

### 2.3. Mobile App Intervention

The digital intervention used in this study was hosted by SilverCloud Health [SilverCloud Health Inc, Boston, MA, USA] and comes in both a desktop and mobile app format. Teens in this study engaged with the Space from Body and Eating Concerns Program (SBEC), which includes six modules based on cognitive-behavior therapy for EDs. The adult version of the Space from Body and Eating Concerns Program was derived directly from an earlier version shown to be effective in reducing clinical symptoms in college and university aged women with EDs [19] and is now being used in a large clinical trial [50]. Modules aim to promote a better understanding of how disordered eating behaviors develop and are maintained and to help users learn strategies for implementing regular eating, identifying and reframing ED-maintaining thoughts, managing feelings and emotions that may trigger ED behaviors, and using coping skills to reduce ED symptom severity. Content adaptations made after the Discovery Group phase were specific to ED symptom manifestations in teens, including wording/image changes consistent with age and reading level, addition of content related to interactions with parents and peers, more information regarding media health and EDs, and reduction of text and video length. The core CBT based interventions were retained. Based also on the high number of sexual minority participants recruited in the Discovery phase and the subsequent decision to expand inclusion criteria to other minority groups including males and gender minorities for the testing phase, content within the app was also adapted after discovery phase to be applicable to teens of all gender identities (e.g., altering “thin-ideal” to “thin or muscular ideal”) and to be more inclusive to sexual minorities (e.g., representation of relationships other than heterosexual in the relationship module and personal stories).

### 2.4. Quantitative Survey Measures

#### 2.4.1. ED Characteristics

The Stanford-Washington Eating Disorder Screen (SWED) was used in this study to assess participant risk of having an ED based on DSM-5 criteria (i.e., possible anorexia nervosa (AN), bulimia nervosa (BN), binge-eating disorder (BED), subclinical BN, subclinical BED, purging disorder, unspecified feeding or eating disorder (UFED), high risk for an ED, and no ED). Eligible participants were grouped into two ED risk groups based on their results; clinical ED (i.e., AN, BN, BED, and purging disorder) or subclinical ED (i.e., subclinical BN, subclinical BED, and UFED), and high risk for an ED. Those who screened positive for AN (not including atypical AN, which is not currently assessed by the SWED) were excluded from this study, as these participants may require more extensive medical monitoring and supports than can be provided via a digital tool. The SWED provides good sensitivity and specificity for most ED diagnoses [43] and has been used among those 13 years and older [51]. Participants also reported on the chronicity of their ED symptoms by responding to the question, “*You indicated that you have been experiencing some concerns related to your eating, shape, or weight. When did you first start experiencing these symptoms?*” with the following response options: Less than one month ago, More than one month ago but less than 3 months, More than 3 months ago, More than 6 months ago, More than 1 year ago, More than 3 years ago, More than 5 years ago.

#### 2.4.2. Mental Health Comorbidities

In addition to ED symptoms, depression and anxiety symptoms were also assessed among teens in this study. Depression symptoms were assessed using the Patient Health Questionnaire (PHQ-9) scale, a nine-item instrument used to measure depression severity [52]. The PHQ-9 score ranges from 0 to 27; scores of less than 5 indicate no depression, scores 5–9 indicate mild depression and scores of greater than 9 indicate moderate to severe depression [53]. Anxiety disorders were assessed using the Screen for Child Anxiety Related Disorders (SCARED) [54]. The scale consists of 41 items in 5 subscales: panic disorder, generalized anxiety disorder, separation anxiety disorder, social anxiety, and significant school avoidance. The SCARED scores range from 0 to 82.

#### 2.4.3. Mental Health and ED Treatment

Survey questions were also included asking about past engagement with treatment for EDs, treatment for other mental health disorders, barriers to ED treatment, and perceived utility of the SBEC Program adapted for teens in reducing ED symptoms.

#### 2.4.4. Social Media Use

We used two questions to measure participant use of nine major social media platforms among our participants. Specifically, these two questions included (1) if they used the social media platform several times a day; (2) if they follow any accounts about the thin-ideal on this social media platform.

#### 2.4.5. Gender and Sexual Identity

We asked participants about gender identity (“*What is your gender identity?*”) with response options of (1) Female, (2) Male, (3) Transgender Man, (4) Transgender Woman, (5) Genderqueer/gender non-conforming, (6) Prefer to self-identify. Those who did not identify as female or male were included as a gender minority. We also asked participants their sexual identity (*“How would you describe your sexual attraction?* Select all that apply.”) with response options of (1) Attracted to men/males/masculinity, (2) Attracted to women/females/femininity, (3) Bisexual, (4) Pansexual, (5) Asexual, (6) Questioning, (7) Prefer to self-identify. Those who indicated in options 1 and 2 for attraction to those of the same identity as their own gender identity, those who indicated response options 3–7, or those who marked multiple responses were included as sexual minority participants. A collapsed category was created for teens who identify as a gender and/or sexual minority and this is labeled as Lesbian, Gay, Bisexual, Transgender, Questioning, + (LGBTQ+) in Table 1. Participants within the Discovery phase did not receive this gender identity question within the survey, as our inclusion criteria was specific to those who identify as female in this preliminary study (see Refinements and Testing Group Methods). 

### 2.5. Statistical Approach

Descriptive statistics were conducted to determine differences in sample characteristics among teens within the Discovery Group and the Testing Group to illustrate the groups reached by our social media outreach methods, both before and after refinements. All analyses were conducted using Stata MP Version 16 (StataCorp, LLC, College Station, TX, USA).

### 2.6. Qualitative Analysis

Teens in the Discovery Group provided responses to open-ended questions on ED treatment readiness, interest in using an mHealth tool, and detailed questions on app content and features. All open-ended survey responses (N = 169) were reviewed and coded by two coders familiar with EDs and digital interventions (EK, MV). A codebook was created and refined utilizing both inductive and deductive methods to capture overall themes within the dataset. Primary themes included *(1) Positive Feedback, (2) Negative Feedback/Suggestions, (3) Motivations/Barriers to App Use*. Subcodes were included with each primary code to capture all relevant clinical themes present. All discrepancies between the initial two coders were then reviewed by a third expert coder (PCR) to finalize master codes for all responses, a third-party resolution method used previously in qualitative research [55].

## 3. Results

### 3.1. Recruitment Strategy Results

**Figure 1:**
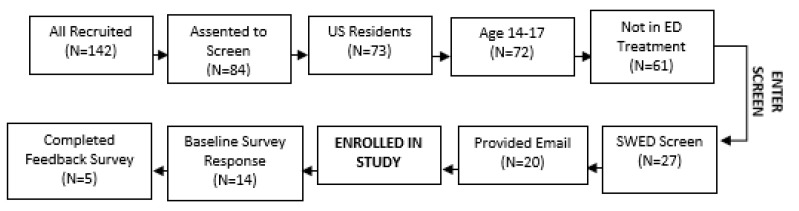
Participants eligible after each online screening step—Discovery Group.

For the Discovery Group, we recruited 142 individuals from Instagram and Facebook over approximately 1 month, 84 of whom provided online assent to continue on to our demographic screener. Within the demographic screening questions, 11 were not US residents, 1 was not within the 14–17 years age range, and 11 were currently in ED treatment. The remaining 61 individuals then entered the SWED screener, with 27 meeting eligibility criteria for EDs (clinical/subclinical or high risk). Of this group, 20 provided contact information and were enrolled in the study. A total of 14 teens completed the baseline survey, with 5 of these teens additionally providing complete quantitative and qualitative feedback on the SBEC-Teen Program. See Figure 1 for screening flow details.

**Figure 2:**
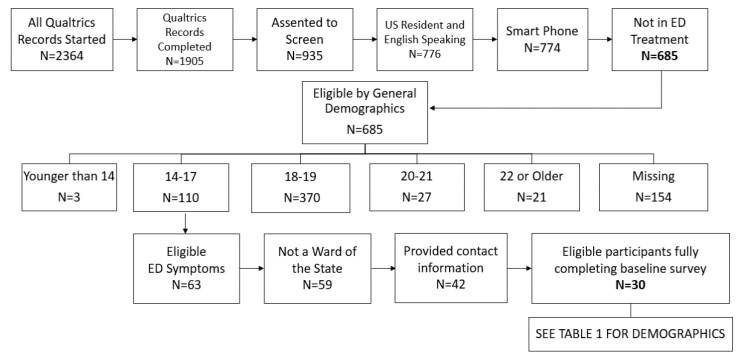
Participants eligible after each online screening step—Testing Phase. Bold numbers indicate those eligible after initial demographic screening questions (N = 685) and those enrolled into the study (N = 30).

**Figure 3:**
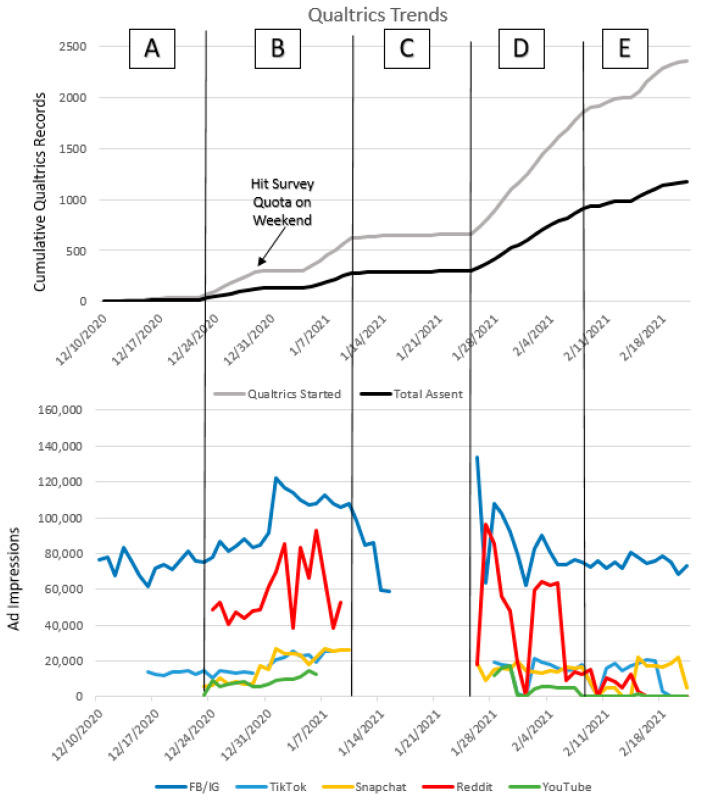
Cumulative Qualtrics records created compared to recruitment platforms used to recruit the Testing Group. (**A**) Facebook/Instagram (FB/IG) only, added in TikTok, (**B**) three additional platforms added, (**C**) paused additional platforms (reversal design), (**D**) all platforms with ads shuffled and more money allocated to Snapchat and Reddit, (**E**) reduced to standard daily budget.

For the Testing Group, 2364 individuals clicked on our advertisements on multiple social media platforms over approximately 2 months and 1905 of these Qualtrics records started were completed. After basic eligibility criteria 685 were still eligible and entered the SWED screener. Among these participants, a majority were 18–19 years old (370, 54%) followed by those 14–17 (110, 16%). We also excluded individuals who do not provide data on core screening questions (e.g., age; N = 154) [56]. See Figure 2 for screening flow details. Details of the platforms used for recruitment are detailed in Figure 3, demonstrating the number of impressions of advertisements on each platform as associated with cumulative Qualtrics records created during each timeframe in the recruitment process. Rate of recruitment began slowly when using only Facebook and Instagram (as done in Discovery phase) so TikTok was added in but produced little change. Reddit, Snapchat, and YouTube were then added and increased the rate of Qualtrics records completed dramatically. We paused our advertisements on Reddit, Snapchat, and YouTube as a means of reversal to verify that these platform additions were the factor causing our recruitment rates to increase (ruling out that it was not just that our Instagram and Facebook ads had gained traction). After this verification, we then shuffled our advertisements on all platforms to ensure those that were most successful were allocated the highest daily budget, which again increased our rate of recruitment. As we neared our 30-participant target sample size for the Testing Group, we reduced our budget to a standard daily amount, which produced steady recruitment until we reached our recruitment goals.

### 3.2. Sample Descriptive Characteristics

Sample characteristics for teens in the Discovery Group and Testing Group are shown in Table 1. Participants were primarily white (92% Discovery Group, 74% Testing Group), and identified as female (100% Discovery Group (due to gender exclusion criteria), 74% Testing Group). Participants in the Discovery Group were distributed evenly across 14–17 years old, while those in the Testing phase were primarily in the older 16–17-year-old age group (83%). A majority of the participants in the Discovery Group identified as a sexual minority (71%), and 63% of teens in the Testing Group identified as being a gender or sexual minority. While only Instagram/Facebook was used for recruitment in the Discovery Group, among those in the Testing Group, Snapchat was the most successful recruitment platform (60%) with Instagram the second most successful (30%); Instagram and Snapchat were also the most commonly used platforms within our sample (see Supplementary Table S3). Within both groups recruited, nearly all participants experienced depression (100% in the Discovery Group, 90% in the Testing Group), and 83% of those in the Testing Group had moderate to severe depression. Anxiety was also very high among this group, with 100% of those in the Discovery phase and 93% in the Testing phase having any anxiety disorder symptoms.

Table 2 outlines details for ED symptom levels, prior engagement with ED or mental health treatment, and ED treatment interest. Results from the SWED screen indicated 64% of the Discovery Group and 67% of the Testing Group had a Clinical or Subclinical ED (not including AN), with remaining participants screening as at risk or high risk for an ED. With regard to prior engagement with treatment, 70% of the Discovery Group and 56% of the Testing Group had engaged with some type of mental health treatment before, and only 10% of both groups had specifically engaged with ED treatment. Those in the Discovery Group reported their biggest barriers to in-person ED treatment was not wanting to tell their parents (30%) about their eating concerns and worrying that treatment would make them emotionally uncomfortable (30%). The Testing Group also reported telling parents about their eating concerns as their biggest barrier to ED treatment (27%). Despite these reported barriers, nearly two thirds of participants in both groups stated they were interested in ED treatment (60% Discovery Group, 67% Testing Group).

### 3.3. Qualitative Results

Supplementary Table S1 outlines the frequency of Discovery Group open-ended responses as well as example participant quotes within each of two primary codes, positive feedback, and negative feedback/suggestions, with regard to the SBEC-Teen Program intervention reviewed. Among the 96 responses (56.8% of total) providing positive feedback on the app, roughly 28.1% were related to participants’ perceptions of the positive impact of the app on ED recovery, 13.5% were related to usability of the app, and 8.3% were related to the app as useful in comparison to in-person ED treatment. Of the 42 responses providing negative feedback/suggestions (24.9% of total), 69.0% involved suggestions centered around improvements for app content (e.g., suggestions for adding/removing topics), 14.3% provided feedback on app presentation (e.g., length of text/videos), and 4.8% gave suggestions on inclusion of gamification or incentives for use.

Supplementary Table S2 outlines motivations and barriers for app use stated by teens. Motivations for use outweighed barriers listed (26 vs. 10), with motivations focused on improvements in ED symptoms, other mental health symptoms, or on overall wellbeing. Barriers to use mentioned by teens included being busy or forgetting to use the app, trying to reduce screen time, not being ready for ED treatment, and depression symptoms.

## 4. Discussion

Teens’ frequent use of SM, especially via smartphone, is a promising avenue for targeted recruitment of at-risk teens for connection with digital treatment. Personalized content, features, and targeted outreach are factors which can increase user adoption and engagement with such tools, which can in turn lead to greater symptom improvement [57,58,59]. As such, the present study aimed to test the feasibility of innovative outreach methods on SM for teens with EDs and to garner feedback from this population to further adapt and tailor an mHealth intervention for this population.

Our SM recruitment strategies in this study successfully identified teens at high risk for EDs and those with subclinical and clinical EDs. Notably, our sample of teens with EDs recruited from social media also reported high levels of other mental health concerns including depression and anxiety. This is in line with previous research noting the high overlap between these mental health disorders and highlights the well-established associations between disordered eating and depression and anxiety symptoms [60]. All participants reported some symptoms of depression in the Discovery Group, with a few noting that low mood or lack of motivation for activities could impact their use of the app [20,61,62]. Additionally, the majority of teens in both the Discovery and Testing Groups endorsed significant anxiety symptoms, which could impact sustained engagement with an mHealth tool. While primary motivations for app use described by the Discovery Group were a desire to specifically reduce ED symptoms, secondary motivators for app use also included a desire to increase wellbeing more generally (e.g., improve mood, quality of life). Indeed, previous research has shown that improvement of ED symptoms can also improve overall wellbeing and functional outcomes [63]. In the future, content and features of digital interventions for EDs among teens could also aim to more directly address comorbid concerns such as depression and anxiety which could contribute to body image and eating concerns and also impact adherence to digital treatment.

This study provided preliminary evidence for and information to refine our social media recruitment approach, adding to the limited literature on social media recruitment, especially for this study’s target population [64,65,66]. For the Discovery Group we effectively recruited from both Facebook and Instagram. To expedite recruitment for the Testing Group we expanded to Snapchat, Reddit, YouTube, and TikTok in order to reach a wider population. Snapchat was found to be the most successful platform for recruitment, and such image-based platforms like Snapchat and TikTok could be an important area of expansion for future research as teens may use these platforms with dynamic and interactive content more often than traditional platforms (e.g., Facebook) [17,67]. Furthermore, these platforms are more heavily image based and include other features like image filters and video editing that may promote social comparisons and the thin ideal making them ideal avenues to recruit teens at risk for EDs [68,69]. Of note, Snapchat has some restrictions on specific language used in targeted advertisements for those under 18 years of age, so the use of general language such as “teens” could have accounted for the large number of 18–19-year-olds completing the screen. We were able to collect data on this age group within our screen which demonstrates the need for and interest in support for ED symptoms among older teens whose symptoms may have progressed to subclinical or clinical levels. Relatedly, an alarming 67% of the teens in the Testing Group reported experiencing ED symptoms for over 3 years, highlighting the need for early assessment and intervention to possibly mitigate ED onset and symptom progression.

Other refinements during the Testing Group phase included expanding recruitment of teens who identified only as female to those of all gender identifies, as informed by the high number of participants who identified as a sexual minority in the Discovery phase. The extremely high percentage of gender and sexual minority participants in the Testing Group (63%) also highlights the potential for SM recruitment to reach diverse populations that are understudied and undertreated, and aligns with emerging research suggesting that individuals from gender and sexual minority groups may experience higher levels of body dysmorphia and other ED symptoms [70,71,72,73,74,75]. Social media outreach may provide an avenue to access these minority groups, who may not yet be ready to disclose either their ED symptoms or their sexual or gender identities to caregivers or health professionals [76]. This fear of disclosure could drastically reduce or delay identification of body image and eating concerns among this group, limiting timely connection with specialized care, and resulting in progression of negative health outcomes. However, digital outreach and mHealth intervention may provide a promising way to reach young teens to provide support without mandating parental consent, which has been shown to be a barrier to care among teens with EDs in this study and in previous literature [36]. However, rates of ethnic and racial minority groups reached in Testing phase were comparable to, or below, national estimates for EDs among these groups [77,78] and it is unclear why such a high level of gender and sexual minority participants were recruited but not ethnic or racial minorities, for which some groups may also experience elevated eating concerns [79,80]. Future investigations should adapt or evaluate targeted advertisement content, including images and keywords used, to improve outreach and connection with ethnic and racial minority teens who may benefit from such digital supports for ED symptoms.

The findings of these studies should be considered in the context of several limitations. First, as these were preliminary studies aimed to pilot and refine an innovative outreach method to reach young teens with EDs and screening criteria was tight to identify those only with binge/purge-type EDs or at high risk for an ED, the final sample size for analysis was limited. However, data generated through usability testing of our innovative social media recruitment methods also allowed us to assess for the needs of different age and demographic groups for such an mHealth intervention. Although the Discovery Group who provided qualitative feedback was small, sample sizes of five participants have been shown to be effective in past literature for identifying 55–99% of digital tool usability issues [81]. The Testing Group, whose baseline demographics are presented here, will evaluate the intervention’s initial feasibility and acceptability, and a sample size of 30 teens is within the range of samples sizes for other pilot tests of digital therapeutics [82,83,84,85,86,87,88]. Due to COVID-19, qualitative interviews for the Discovery Group were adapted for completion via survey format, which may have reduced response rates due to the number of open-ended questions.

## 5. Conclusions

Overall, teens with EDs in the Discovery Group mentioned high acceptability and interest in the intervention and suggested including more content about the risks of EDs and incorporating more interactive components such as rewards, gamification, or incentives for app use, which have been found to impact use of digital interventions among teens and adolescents in previous research [89]. End user feedback is critical for the iterative refinement of digital interventions such as the SBEC-Teen Program, especially among young teens for whom there are few tailored digital interventions for ED recovery support and may influence engagement levels and subsequent efficacy of such interventions. Outreach efforts and intervention content specific to the needs of minority groups who may otherwise be reluctant to access in-person care due to lack of specialty care or stigma is important, as is the inclusion of efforts to proactively identify young teens with subclinical EDs who may not be aware of the clinical severity of their symptoms or who have not yet accessed any form of mental health care. Further, during the COVID-19 pandemic, alterations to daily routines and school schedules, increased stress, and changes to food access and social interactions have recently been linked to increases in ED symptoms [90,91]. Especially given the limitations and/or disruptions to in-person care during this time, digital interventions can help to provide supports either prior to entry into in-person ED care or to bridge the gap between appointments. Furthermore, recruitment via social media allows for targeted outreach to teens struggling with EDs, including those with comorbid mental health concerns and from at-risk minority groups, and connection of these groups with digital interventions aimed to improve ED symptoms and possibly increase motivations for in-person recovery is feasible and promising.

## Figures and Tables

**Table 1:** Discovery and Testing Group sample characteristics.

	Discovery Group (N = 14)	Testing Group (N = 30)
	N (%)	N (%)
***Age***		
14–15	7 (50%)	5 (17%)
16–17	7 (50%)	25 (83%)
***Race***		
White	11 (92%)	22 (74%)
Other	2 (8%)	8 (26%)
***Ethnicity***		
Hispanic	2 (14%)	4 (13%)
Non-Hispanic	12 (86%)	26 (87%)
***Gender***		
Female	14 (100%) ^a^	22 (74%)
Male	-	4 (13%)
Gender Minority	-	4 (13%)
***Sexual orientation***		
Heterosexual	4 (29%)	13 (43%)
Sexual Minority	10 (71%)	17 (57%)
***LGBTQ + Group***	-	19 (63%)
***Platform Recruited From***		
Instagram/Facebook	14 (100%)	6 (30%) ^b^
Snapchat	-	12 (60%)
YouTube	-	1 (5%)
TikTok	-	1 (5%)
Reddit	-	0
***Mental Health Comorbidities***		
**Depression**		
No depression	0	3 (10%)
Mild depression	2 (20%)	2 (7%)
Moderate/Severe depression	8 (80%)	24 (83%)
**Anxiety**		
Any anxiety	10 (100%)	27 (93%) ^c^
Panic Disorder	9 (90%)	22 (76%)
Generalized Anxiety Disorder	3 (30%)	25 (89%)
Separation Anxiety	6 (60%)	18 (62%)
Social Anxiety Disorder	7 (70%)	15 (54%)
School Avoidance	0	23 (79%)

^a^ Inclusion criteria for Discovery Group recruitment only included those who identified as female. ^b^ Testing group recruitment platform sample size is 20 (question added halfway through). ^c^ Testing group sample sizes vary for anxiety due to missing data.

**Table 2:** Discovery and Testing Group ED symptoms and treatment.

	Discovery Group N = 14 ^a^	Testing Group N = 30
	N (%)	N (%)
***ED symptoms***		
Clinical/Subclinical ED (not including AN)	9 (64%)	20 (67%)
High risk ED	5 (36%)	10 (33%)
***ED chronicity***		
More than 3 years	5 (50%) ^a^	20 (67%)
Less than 3 years	5 (50%)	10 (33%)
***Prior ED treatment***	1 (10%)	3 (10%)
***Prior mental health treatment***	7 (70%)	17 (56%)
***Largest ED treatment barrier***		
I do not want to tell my parents about my eating concerns	3 (30%)	8 (27%)
I do not want to tell a healthcare professional about my eating concerns	1 (10%)	1 (3%)
I am too busy for treatment	2 (20%)	3 (10%)
I am worried treatment would make me emotionally uncomfortable	3 (30%)	3 (10%)
I am not interested in treatment	1 (10%)	4 (13%)
***Interested in ED treatment***	6 (60%)	20 (67%)
***Think such mHealth app would be useful in reducing ED symptoms***	6 (50%)	13 (43%)

^a^ Discovery group sample size was *n* = 10 for all measures excluding SWED (*n* = 14) and Perceived Treatment Usefulness (*n* = 12).

## Data Availability

The data presented in this study are available on request from the corresponding author. The data are not publicly available as this is a preliminary and usability study.

## Supplementary Materials

The following are available online at https://www.mdpi.com/article/10.3390/ijerph18157979/s1, Table S1: Discovery Group Participant Feedback on App Content and Features, Table S2: Discovery Group- Teen Motivations and Barriers for app engagement, Table S3: Social Media Use Among Those in Discovery and Testing Groups.
