# Supplementary material for: Exploring Social Media Recruitment Strategies and Preliminary Acceptability of an mHealth Tool for Teens with Eating Disorders
Source: Int J Environ Res Public Health. 2021 Jul 28;18(15):7979. doi: 10.3390/ijerph18157979 (PMC8345665; doi:10.3390/ijerph18157979)
Supplement: Supplementary file 1 [file ijerph-18-07979-s001.zip › ijerph-1284382-supplementary.pdf]

Supplementary Materials: The following are supplementary items to go online: Supplementary Table S1, Supplementary Table S2, Supplementary Table S3.

Supplementary Table S1. Discovery Group Participant Feedback on App Content and Features

Theme	N (%)	Participant Quotations
**Positive Feedback**	96 (56.8%)[a]	• *"There's so many resources, it could definitely help me through a tough time"*<br>• *"It provides healthier alternatives to destructive coping mechanisms."*<br>• *"Absolutely, it allowed me to easily pick up on triggering situations so that I could avoid them."*<br>• *"I found the app helpful and I actually learned a bunch of good information from it."*
--- ED Specific	27 (28.1%)[b]	• *"I found that the app provided helpful strategies in managing my eating disorder."*<br>• *"It definitely has changed my behaviors. i've been tracking my eating and just overall being healthier with food."*<br>• *"I think that it can help me to become more mindful of my behaviors and thoughts towards eating and my body"*<br>• *"for sure, it already started to have a positive impact on my body image and eating behaviors."*<br>• *"Help with binging/purging urges and help with convincing myself to eat/staying on track with meal plans"*
--- Ease of Use/Accessibility	13 (13.5%)[b]	• *"thinking well and body wellness. those really helped me gain confidence and helped me take control of something i thought was out of my reach."*<br>• *"it seems like it has a lot of potential in helping teens who struggles with eating disorders."*<br>• *"i honestly do not feel like it was very difficult to navigate. it was fairly simple, and i feel like tons of people would love the simplicity of the app."*<br>• *"the fact that i had such a convenient way to better myself."*
--- Compare to In-Person Treatment	8 (8.3%)[b]	• *"It's easier to do so in a more 'anonymous' way for me than in person. It's less shameful."*<br>• *"it is less personal."*<br>• *"some people get too scared or just can't afford clinical help, or just might be afraid to tell their doctors or therapists. This can help alleviate those fears and get them the help they need."*

**Negative Feedback/Suggestions for Improvement**	42 (24.9%)[a]	• *"this app is honestly better for people who aren't that deep in disordered eating yet."*<br>• *"It's not enough for me and I'm also not ready for recovery."*
--- App Content	29 (69.0%)[c]	• *"Definitely include more information (especially concerning/including different type of ED's and their risks)"*<br>• *"I would make the modules shorter and more to the point"*<br>• *"They're vague and I feel like there could be better goal setting skills enforced"*<br>• *"it could be improved by expanding on healthy food choices teens can make"*
--- Presentation of App Content	6 (14.3%)[c]	• *"Make it more engaging especially visually."*<br>• *"make the layout more simple and user friendly."*
---Gamification	2 (4.8%)	• *"maybe a reward/point system. It doesn't have to be real incentives, maybe just like some form of earning points or cute badges for completing goals/modules."*
---Incentives/Reminders to Use	2 (4.8%)[c]	• *"I would probably not touch this app at all since there's not much incentive/motivation to use it especially since I'm busy and often times forget about mental health."*

[a]Percentages out of total excerpts (N=169)
[b]Percentages out of overall Positive Feedback responses (N= 96)
[c]Percentages out of overall Negative Feedback responses (N=42)

Supplementary Table S2. Discovery Group- Teen Motivations and Barriers for App Engagement

Theme	N (%)	Participant Quotations
**Motivations for App Use**	26 (15.4%)[a]	
--- ED Recovery	5 (19.2%)[b]	• *"Wanting to fully recover from my eating disorder."*<br>• *"I have found it helpful in managing my eating disorder thoughts and behaviors"*
---MentalHealth/ Wellbeing	7 (26.9%)[b]	• *"Yes! This is the most helpful app i've tried to help with my wellness!"*<br>• *"yeah, the app helped me to focus on myself and my personal well being. being a teen, it is hard to put yourself first sometimes, and the app really helped me to do so."*
**Barriers to App Use**	10 (6.0%)[a]	
**---** Busy/forget	4 (40.0%)[c]	• *"constantly running around with sports, schooling, and other activities."*<br>• *"I would probably not touch this app at all since there's not much incentive/motivation to use it especially since I'm busy and often times forget about mental health."*
--- Reducing Screen time	2 (20.0%)[c]	• *"I am trying to spend more time away from the screen and focusing on more important things in my life."*
--- Not ready for Recovery	3 (30.0%)[c]	• *"If I were accepting of recovery, I would see benefit in going through the exercises/assignments, though without continued practice or personalized therapy, I would very easily relapse"*<br>• *"i struggle with depression, so i do have days where i don't do much at all, so days like that & just forgetting to get onto the app, really."*<br>• *"Probably business, and my depression which causes me to lose interest in nearly everything. It's hard to keep interest or motivation to do something repeatedly."*
**---** Depression	2 (20.0%)[c]	

[a]Percentages out of total excerpts (N=169)
[b]Percentages out of overall Motivations for App Use responses (N= 26)
[c]Percentages out of overall Barriers to App Use responses (N= 10)

Supplementary Table S3. Social Media Use Among Those in Discovery and Testing Groups

	Study 1	Study 2
	N=14	*N=30*
Instagram		
Use several times a day[a]	11 (79%)	20 (69%)
Follow any accounts about thin-ideal	11 (84%)	21 (70%)
Snapchat		
Use several times a day	8 (57%)	17 (57%)
Follow any accounts about thin-ideal	4 (31%)	12 (41%)
Twitter		
Use several times a day	3 (21%)	4 (13%)
Follow any accounts about thin-ideal	2 (15%)	4 (14%)
Facebook		
Use several times a day	1 (7%)	4 (14%)
Follow any accounts about thin-ideal	1 (7%)	5 (17%
YouTube		
Use several times a day	3 (21%)	7 (23%)
Follow any accounts about thin-ideal	6 (46%)	12 (40%)
Pinterest		
Use several times a day	0	2 (7%)
Follow any accounts about thin-ideal	4 (33%)	7 (25%)
TikTok		
Use several times a day	-	12 (40%)
Follow any accounts about thin-ideal	-	13 (43%)
Reddit		
Use several times a day	0	0
Follow any accounts about thin-ideal	1 (8%)	2 (7%)
Tumblr		
Use several times a day	0	0
Follow any accounts about thin-ideal	3 (23%)	3 (10%)

[a]Within the Discovery Group 14 participants responded to social media usage questions and 13 people responded to following any accounts with thin-ideal content
